# EHMTI-0269. The idiopathic intracranial hypertension without papilloedema counterfeits the migrainous headache in clinical presentation

**DOI:** 10.1186/1129-2377-15-S1-C37

**Published:** 2014-09-18

**Authors:** S Ljubisavljevic, J Zidverc-Trajkovic

**Affiliations:** 1 Clinic for Neurology, Clinical center Nis, Nis, Serbia; 2 Clinic for Neurology, Clinical center Serbia, Belgrade, Serbia

## Introduction

Idiopathic intracranial hypertension (IIH) is a pathological state defined as an increase of intracranial pressure in the absence of a causative pathological process.

## Aim

To evaluate the clinical features of the patients with IIH diagnosed in our Headache Ambulances according to the current knowledge of this disorder.

## Method

In the retrospective and cross-sectional analysis of 3550 patients we present 18 newly diagnosed IIH patients, 15 women and 3 men, aged from 18 to 51, with obtained values of cerebrospinal fluid pressure between 220 and 680 mm of water. The symptoms of IIH clinical presentation have been headache, reported by 94 % of patients without optic nerve papilloedema (PE) noted in 30 % of them.

## Results

The obtained results confirmed the presence of headache features that are included in criteria for headache attributed with IIH in patients with PE. On the other side, it is worth to note, that in patients without PE majority of them (80%), had ‘‘migrainous’’ features of headache and accompanying symptoms.

## Conclusion

We assume that a misdiagnosis of ‘‘primary’’ chronic migraine might be possible in patients without PE who may in fact suffer from a headache attributed to IIH according to currently used ICHD criteria. It seems that the headache profile of IIH is not pathognomonic as it sometimes mimics the primary headaches. We could conclude that some chronic migraine patients refractory for treatment might be patients with IIH.

No conflict of interest.

